# Phase I study of TAS-115, a novel oral multi-kinase inhibitor, in patients with advanced solid tumors

**DOI:** 10.1007/s10637-019-00859-4

**Published:** 2019-12-10

**Authors:** Toshihiko Doi, Nobuaki Matsubara, Akira Kawai, Norifumi Naka, Shunji Takahashi, Hiroji Uemura, Noboru Yamamoto

**Affiliations:** 1 Department of Gastrointestinal Oncology, National Cancer Center Hospital East, 6-5-1 Kashiwanoha, Kashiwa, Chiba, 277-8577 Japan; 2 Department of Breast and Medical Oncology, National Cancer Center Hospital East, 6-5-1 Kashiwanoha, Kashiwa, Chiba, 277-8577 Japan; 3 Musculoskeletal Oncology and Rehabilitation, National Cancer Center Hospital, 5-1-1 Tsukiji, Chuo-ku, Tokyo, 104-0045 Japan; 4 Orthopedics (Bone Soft Part Tumor Department), Osaka International Cancer Institute, 3-1-69 Otemae, Chuo-ku, Osaka, Osaka 541-8567 Japan; 5 Medical Oncology, The Cancer Institute Hospital, JFCR, 3-8-31 Ariake, Koto-ku, Tokyo, 135-8550 Japan; 6 Urology and Renal Transplantation, Yokohama City University Medical Center, 4-57 Urafune-cho, Minami-ku, Yokohama, Kanagawa 232-0024 Japan; 7 Department of Experimental Therapeutics, National Cancer Center Hospital, 5-1-1 Tsukiji, Chuo-ku, Tokyo, 104-0045 Japan

**Keywords:** TAS-115, Phase 1, Multi-kinase inhibitor, Solid tumors, MET, VEGFR, FMS, PDGFR

## Abstract

TAS-115 is a novel MET, VEGFR, FMS and PDGFR inhibitor, developed to improve the continuity of drug administration with a relatively short half-life. We assessed its tolerability, safety, pharmacokinetics, efficacy, and pharmacodynamics in patients with solid tumors. This open-label, dose-escalation phase I study of TAS-115 consisted of three parts: part 1 (TAS-115 was administered orally once daily [SID]); part 2 and an expansion part (SID in a 5 days on/2 days off [5-on/2-off] schedule for 21 days per cycle). In part 1 (200–800 mg SID administered to 21 patients), systemic exposure after single administration increased almost dose-proportionally. Three dose-limiting toxicities (DLTs) were observed in three patients: grade 3 rash (650 mg), thrombocytopenia with bleeding, and rash (800 mg). The maximum tolerated dose (MTD) was determined as 650 mg SID. In part 2, the 5-on/2-off schedule was evaluated at the MTD to improve treatment exposure. No DLTs were observed and no patients required treatment interruption in cycle 1. During part 2 and the expansion part (*N* = 61), grade ≥3 treatment-related adverse events were reported in 47 patients, with neutropenia (24.6%), hypophosphatemia (21.3%), anemia, and thrombocytopenia (14.8% each), and leukocytopenia (11.5%) occurring in ≥10% of patients. The best overall response was stable disease in 31 of 82 patients (37.8%). An apparent reduction in fluorodesoxyglucose-uptake and bone scan index was observed in some patients. TAS-115 was generally well tolerated, with manageable toxicities and recommended phase II dose was estimated as 650 mg SID, 5-on/2-off. Furthermore, promising antitumor activity was observed.

**Electronic supplementary material:**

The online version of this article (10.1007/s10637-019-00859-4) contains supplementary material, which is available to authorized users.

## Introduction

Vascular endothelial growth factor (VEGF)-mediated angiogenesis is initiated by the binding of VEGF to the specific VEGF receptor (VEGFR) on vascular endothelial cells. VEGFR is subsequently activated via autophosphorylation by tyrosine kinase in the intracellular domain, resulting in signal transduction for cell growth or other events [1]. To date, the blockade of VEGF/VEGFR signal transduction pathways has been demonstrated to inhibit tumor angiogenesis, which leads to the suppression of tumor growth [2].

It has been demonstrated that the binding of hepatocyte growth factor (HGF), a growth factor secreted from tumor cells or surrounding interstitial tissue, to the HGF receptor (MET) induces the autophosphorylation of MET. This then leads to the activation of intracellular signaling cascades involving mitogen-activated protein kinase (MAPK) and phosphatidylinositol 3-kinase (PI3K)/AKT, which in turn promotes proliferation, migration, invasion, and tubulogenesis [3]. In addition, hypoxia in tumor tissues caused by the inhibition of VEGFR has been reported to induce the expression of MET and contribute to the resistance to VEGFR inhibition [4]. McDonough feline sarcoma (FMS), also called colony-stimulating factor-1 receptor (CSF-1R), is a transcription product of the proto-oncogene c-fms [5]. Its ligand CSF-1 (also known as macrophage [M]-CSF) is a hematopoietic growth factor in the mononuclear phagocyte system involved in the differentiation, proliferation, and survival of macrophages [6, 7]. Macrophages exist in various tissues in the body with their known functional diversity and typically include microglia in the brain, osteoclasts involved in bone remodeling, and Kupffer cells participating in lipid metabolism and detoxification in the liver. The abnormal activation of these macrophages causes various disease conditions [8], including increased function of osteoclasts in bone metastases [9]. Platelet-derived growth factor (PDGF), a growth factor primarily involved in migration and proliferation of mesenchymal cells such as fibroblasts, has been reported to participate in various angiogenic processes [10]. Fibroblasts have recently attracted attention for their roles in tumor growth [11, 12].

TAS-115, a novel oral multi-kinase inhibitor, inhibits the autophosphorylation of MET, VEGFR, FMS, PDGF receptor (PDGFR) and other receptors in an adenosine triphosphate (ATP)-competitive manner. The IC_50_ values of TAS-115 against recombinant VEGFR2 and recombinant MET were 0.030 and 0.032 μmol/L, respectively. In a 192-panel kinase assay, TAS-115 exhibited an IC_50_ of <1 μmol/L for 53 (28%) kinases, including MET, AXL, c-kit, Src, PDGFR-alpha and –beta [13]. In mouse xenograft models, TAS-115 demonstrated a potent antitumor effect, inhibiting tumor growth and abnormal bone remodeling [13–15].

The phase I study (JapicCTI-111645) was initially conducted using a tablet formulation of TAS-115. Ten patients were evaluated and no dose-limiting toxicities (DLTs) were observed at 6 dose levels (100, 200, 400, 800, and 1200 mg once daily [SID], and 400 mg twice daily [BID]). The absorption of TAS-115 was saturated between 100 and 1200 mg. Therefore, it was concluded that the maximum tolerated dose (MTD) could not be determined for the formulation (data on file).

Here, we report an ongoing phase I study, using the granule formulation of TAS-115, to investigate the safety, tolerability, pharmacokinetics (PK), MTD, recommended phase II dose (RP2D), and optimal administration schedule for TAS-115 in patients with advanced solid tumors. Antitumor activity and pharmacodynamics were also evaluated by quantification of changes in pharmacodynamic markers.

## Patients and methods

### Eligibility

Key inclusion criteria included histologically or cytologically confirmed advanced solid tumors, refractory to standard treatment or with no available standard therapy, age ≥20 years (≥15 years for malignant bone tumors), Eastern Cooperative Oncology Group (ECOG) performance status (PS) 0 or 1, ability to take medications orally, adequate organ function (white blood cell count ≤10,000/mm^3^, neutrophil count ≥1,500/mm^3^, hemoglobin ≥9.0 g/dL, platelet count ≥75,000/mm^3^, serum total bilirubin ≤1.5 mg/dL, serum aspartate aminotransferase [AST] <100 U/L and serum alanine aminotransferase [ALT] <100 U/L, even if serum AST and serum ALT values did not meet the criteria, values of up to 200 U/L or less were acceptable when they were considered to be attributed to the primary disease, estimated creatinine clearance ≥50 mL/min (as per Cockcroft-Gault formula), and life expectancy ≥60 days. Key exclusion criteria included clinically significant heart disease, >grade 1 pre-existing adverse events (AEs) based on Common Terminology Criteria Adverse Events (CTCAE) Version 4.03 [16], brain metastasis with clinical symptoms or requiring treatment, and serious complications.

### Study design and treatment

This phase I study (JapicCTI-132333) began on December 1, 2013, and is currently ongoing. This is an open-label, nonrandomized, dose-escalation study conducted at five sites in Japan. The study has three parts: a dose-escalation cohort using a traditional 3 + 3 design (part 1), a dosing schedule investigation cohort (part 2), and an expansion cohort to assess the safety at the MTD or lower doses among more patients (expansion part). TAS-115 was orally administered SID for 21 days per cycle in part 1, and on a 5 days on/2 days off (5-on/2-off) schedule in part 2 and the expansion part (Supplementary Fig. S1).

For each dose level in part 1, three to six patients were enrolled. DLTs were evaluated during the first cycle. As the starting dose in the dose-escalation phase, 200 mg/day was selected, which was decided based on the previous phase I study using TAS-115 tablets and a preclinical study in dogs using a tablet and granule formulations (both, data on file). The study drug was orally administered under empty stomach conditions (1 h before or 2 h after a meal) with a cup of water. In part 2, the TAS-115 dose ≤MTD was administered SID or BID using a 5-on/2-off schedule. In the expansion part, ≤MTD was investigated for exposure and safety. Based on the efficacy among initially treated patients with bone lesions, we focused on enrolling patients with bone metastases or osteosarcoma in the expansion part. Additionally, castration-resistant prostate cancer (CRPC) patients with bone metastases were treated with TAS-115 450 mg/day because five of six CRPC patients who had received 650 mg/day in the early expansion part had AEs requiring dose reduction.

All patients in parts 1 and 2 were required to be hospitalized for cycle 1 in order to observe AE. Patients continued to receive the study drug until disease progression or intolerable toxicity. TAS-115 dose interruption criteria included neutrophil count <500/mm^3^, platelet count <50,000/mm^3^, grade ≥3 nonhematologic toxicity, and any toxicity at the investigator’s discretion. Patients with continuous dose interruptions for ≥22 days discontinued treatment, except in cases where the clinical benefit of TAS-115 was strongly suspected. A treatment-related AE (TRAE) that fell under DLT occurred during the treatment period required the dose reduction of TAS-115. If a grade ≥2 event or continuing grade 1 event of decreased appetite, malaise, nausea or vomiting which might affect the treatment continuation occurred, the investigator or sub-investigator had to consider the dose reduction. The range of dose reduction was 25% of the dose that led to the decision of dose reduction. The number of dose reductions was up to 2, and the minimum dose was 100 mg/day.

Dose resumption and elevation criteria are shown in Supplementary Table S1.

### Purpose and assessments

The primary objective was to determine the MTD and RP2D of TAS-115 in patients with advanced solid tumors. The MTD was determined based on DLTs during the first cycle and defined as the highest dose where DLT incidence was <33%. DLTs were defined as grade 4 neutropenia >7 days despite G-CSF use; grade 4 febrile neutropenia (≥38.3 °C [101 °F] of >24-h duration); grade 3 or 4 decrease in platelet count associated with bleeding or grade 4 decrease in platelet count of >7 days duration and not resolved with blood transfusion; grade 3 or 4 nonhematologic toxicity not responding to supportive care; or toxicity requiring discontinuation for >7 days.

The secondary objectives were to investigate the PK profile, safety, tolerability, antitumor activity, and pharmacodynamic profile of TAS-115. AEs were graded according to the CTCAE Version 4.03. Response was assessed by radiologic imaging at 6 and 12 weeks from the first dose, and every 12 weeks thereafter. Antitumor effect was evaluated by investigators using Response Evaluation Criteria in Solid Tumors (RECIST) Version 1.1 [17]. If a patient had bone lesions, evaluation was performed using bone scintigraphy and bone scan index (BSI) were calculated by BONENAVI® software (FUJIFILM Toyama Chemical, Co., Ltd., Japan). BSI response was defined as ≥30% reduction in BSI [18]. An exploratory objective was to assess changes in protein levels of soluble MET (sMET), VEGF, and soluble VEGFR2 (sVEGFR2) in plasma, and HGF in serum as pharmacodynamic markers.

### Pharmacokinetic and pharmacodynamic sampling

Blood samples were collected for plasma PK assessments pre-dosing and at 1, 2, 3, 4, 6, 8, 12, and 24 h after TAS-115 administration on Days 1 and 8 of cycle 1 in part 1, and on Days 1, 8, and 19 of cycle 1 in part 2 (only pre-dosing on Day 8). TAS-115 concentrations were measured by validated liquid chromatography-tandem mass spectrometry. A preliminary assessment of the effect of food on the PK profile was performed. Blood samples after administration under fed and empty stomach conditions were collected from the first six patients in the expansion part. Pharmacokinetic parameters were determined using non-compartmental analyses (Phoenix® WinNonlin® version 6.4; Certara LP, Princeton, NJ, USA).

For pharmacodynamic analyses, blood samples were collected on Days 1 and 22 (before dosing, each) in part 1, and Days 1 (pre-dosing) and 19 (pre- and post 6 h) in part 2 and the expansion part. Patients provided written informed consent to provide blood samples for exploratory pharmacodynamics assessments.

### Statistics

The planned sample size was up to 90 patients, and it was not based on the formal calculation because of experimental phase I trial. For pharmacodynamics parameters, *P* values were calculated using the paired t-test for measured values from merged doses. The data cut-off date for the analyses presented in this report was August 19, 2018. This study is registered with Japan Pharmaceutical Information Center Clinical Trial registry, number JapicCTI-132333.

## Results

### Patient disposition and characteristics

Sixty-seven patients received treatment until the cut-off date. A total of 21 patients (three patients for each of the 200, 300, and 450 mg cohorts, and six patients for each of the 650 and 800 mg cohorts) were treated and evaluable for DLTs in part 1. Based on DLTs in cycle 1 of part 1, six patients subsequently received 650 mg SID (MTD) in part 2. In the expansion part, 46 patients received 650 mg SID and nine CRPC patients received 450 mg SID using a 5-on/2-off schedule up to 21 days per cycle. Baseline characteristics of patients are presented in Table 1.

**Table 1:** Baseline characteristics of patients (*N* = 82)

	Part 1						Part 2 + Expansion part	Expansion part	Part 2 + Expansion part
	200 mg/day	300 mg/day	450 mg/day	650 mg/day	800 mg/day	200–800 mg/day Total	650 mg/day	450 mg/day	650 mg/day or 450 mg/day
	SID	SID	SID	SID	SID	SID	5-on/2-off	5-on/2-off	5-on/2-off
	(*N* = 3)	(*N* = 3)	(*N* = 3)	(*N* = 6)	(*N* = 6)	(*N* = 21)	(*N* = 52)	(*N* = 9)	(*N* = 61)
	N (%)	N (%)	N (%)	N (%)	N (%)	N (%)	N (%)	N (%)	N (%)
Gender									
Male	0 (0.0)	1 (33.3)	3 (100.0)	6 (100.0)	5 (83.3)	15 (71.4)	29 (55.8)	9 (100.0)	38 (62.3)
Female	3 (100.0)	2 (66.7)	0 (0.0)	0 (0.0)	1 (16.7)	6 (28.6)	23 (44.2)	0 (0.0)	23 (37.7)
Age (years)									
Median	56	63	56	63	65.5	64	48.5	72	51
Range [Min, Max]	[55, 68]	[48, 76]	[55, 72]	[33, 73]	[55, 70]	[33, 76]	[16, 82]	[46, 80]	[16, 82]
ECOG PS									
0	0 (0.0)	1 (33.3)	3 (100.0)	6 (100.0)	5 (83.3)	15 (71.4)	32 (61.5)	4 (44.4)	36 (59.0)
1	3 (100.0)	2 (66.7)	0 (0.0)	0 (0.0)	1 (16.7)	6 (28.6)	20 (38.5)	5 (55.6)	25 (41.0)
Type of carcinoma									
Gastric/GE junction	0 (0.0)	0 (0.0)	0 (0.0)	3 (50.0)	2 (33.3)	5 (23.8)	0 (0.0)	0 (0.0)	0 (0.0)
Breast	0 (0.0)	1 (33.3)	0 (0.0)	0 (0.0)	1 (16.7)	2 (9.5)	4 (7.7)	0 (0.0)	4 (6.6)
Colorectum	2 (66.7)	0 (0.0)	0 (0.0)	0 (0.0)	0 (0.0)	2 (9.5)	0 (0.0)	0 (0.0)	0 (0.0)
Lung	0 (0.0)	0 (0.0)	0 (0.0)	0 (0.0)	0 (0.0)	0 (0.0)	3 (5.8)	0 (0.0)	3 (4.9)
Prostate	0 (0.0)	0 (0.0)	0 (0.0)	0 (0.0)	0 (0.0)	0 (0.0)	6 (11.5)	9 (100.0)	15 (24.6)
Bladder	0 (0.0)	1 (33.3)	0 (0.0)	0 (0.0)	0 (0.0)	1 (4.8)	5 (9.6)	0 (0.0)	5 (8.2)
Osteosarcoma	0 (0.0)	0 (0.0)	0 (0.0)	0 (0.0)	0 (0.0)	0 (0.0)	20 (38.5)	0 (0.0)	20 (32.8)
Other	1 (33.3)	1 (33.3)	3 (100.0)	3 (50.0)	3 (50.0)	11 (52.4)	14 (26.9)	0 (0.0)	14 (23.0)
Prior chemotherapy									
Yes	3 (100.0)	3 (100.0)	3 (100.0)	6 (100.0)	6 (100.0)	21 (100.0)	46 (88.5)	9 (100.0)	55 (90.2)
Containing VEGF or VEGFR inhibitor therapeutics									
No	1 (33.3)	2 (66.7)	1 (33.3)	4 (66.7)	5 (83.3)	13 (61.9)	38 (73.1)	9 (100.0)	47 (77.0)
Yes	2 (66.7)	1 (33.3)	2 (66.7)	2 (33.3)	1 (16.7)	8 (38.1)	14 (26.9)	0 (0.0)	14 (23.0)

*ECOG PS* Eastern Cooperative Oncology Group performance status, *GE* gastroesophagea, *SID* once daily, *VEGFR* vascular endothelial growth factor receptor

### Determination of MTD and RP2D

Overall, three DLTs were reported: grade 3 rash in one patient who received 650 mg SID, and grade 3 thrombocytopenia with bleeding and grade 3 rash each in one patient receiving 800 mg SID. Therefore, the MTD was determined as 650 mg in part 1. However, all patients who received 650 mg SID required treatment interruption during cycle 1 because of AEs (hypophosphatemia, decreased appetite, fibrin D dimer increased, and rash).

When the 650 mg SID dose was repeated using a 5-on/2-off schedule with the aim of improving administration sustainability in part 2, no DLTs were reported, and no patients required treatment interruption in cycle 1. Furthermore, the median relative dose intensity improved from 58% (part 1 level 4) to 100% (part 2) for the 650 mg dose level during cycle 1. The RP2D was estimated as 650 mg 5-on/2-off for 21 days per cycle.

### Pharmacokinetic profile

**Fig. 1:**
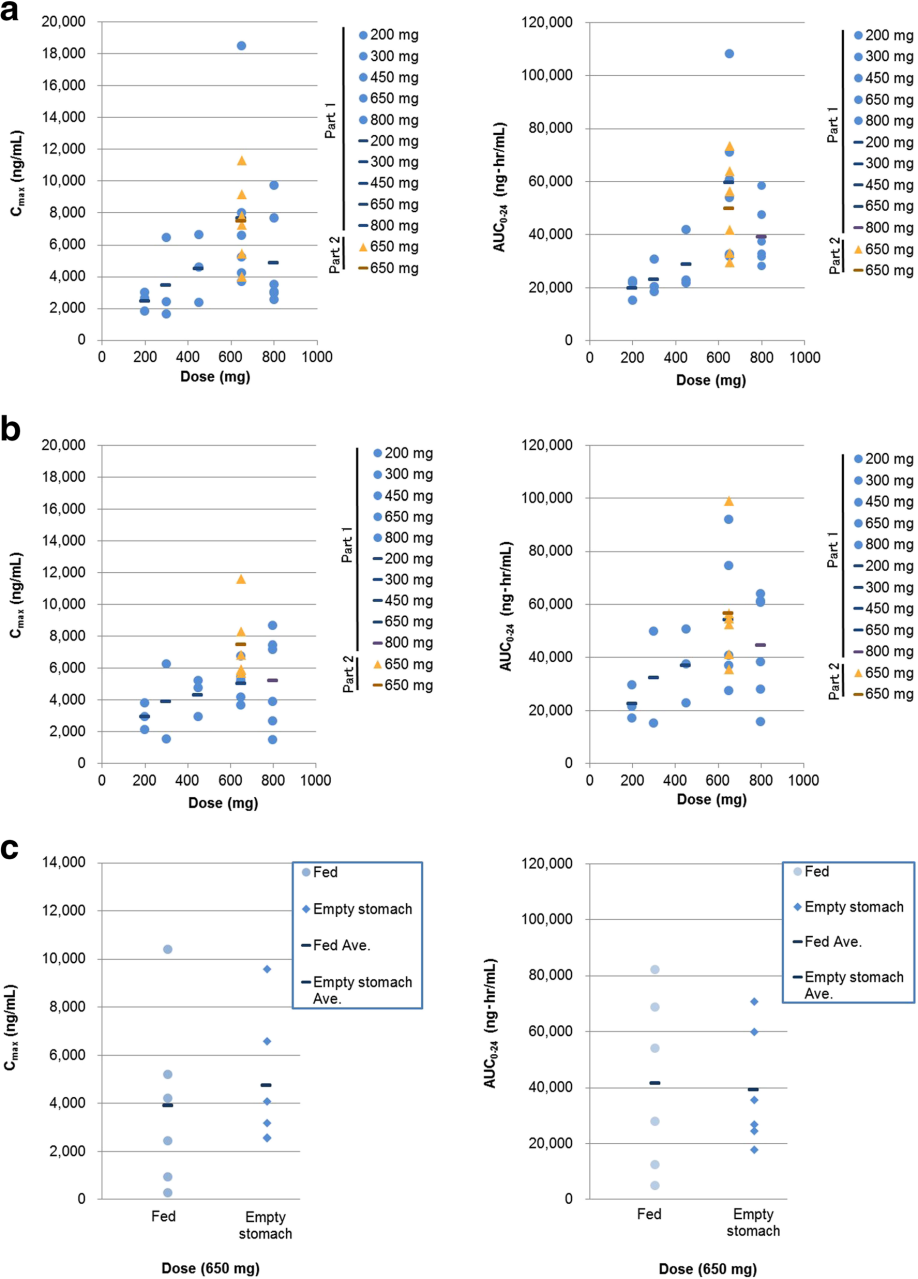
Correlation of C_max_ and AUC_0–24_ with TAS-115 dose on **a** Day 1 and **b** Day 8 (in part 1) or Day 19 (in part 2 and the expansion part), and **c** preliminary assessment of food effect on the pharmacokinetics of TAS-115 (in the expansion part). Each plot of a circle, triangle, or square indicates a particular patient in parts 1 and 2, and a bar indicates the mean value at each dose level. *AUC*_*0–24*_ area under the time-concentration curve from 0 to 24 h, *C*_*max*_ maximum plasma concentration

TAS-115 was rapidly absorbed after oral administration, with the median time to maximum concentration (t_max_) of 1.0–2.0 h. The area under the curve from 0 to 24 h (AUC_0–24_) after single administration increased dose-proportionally over the tested range from 200 mg to 650 mg in part 1, although dose-dependent increase between 650 mg and 800 mg was not seen. The 800 mg dose is only approximately 1.2 times the 650 mg dose, and there were large variations between individual patients (Fig. 1a and Supplementary Table S2). Multiple dosing of TAS-115 for 8 days results in no accumulation in part 1 (Supplementary Table S2). In part 2, the exposure of TAS-115 after multiple administration was similar to the dose level of 650 mg in part 1 (Fig. 1b). The mean and individual PK parameters (maximum plasma concentration [C_max_], and AUC_0–24_) under empty stomach and fed condition were shown in Fig. 1c. The geometric mean ratios (90% CI) of the C_max_ and AUC_0–24_ under fed condition to those under empty stomach condition were 0.545 (0.243–1.224) and 0.812 (0.414–1.593), respectively, however, there were no statistically significant differences.

### Safety

The most common TRAEs were laboratory abnormalities, gastrointestinal symptoms, general disorders, and skin disorders. In part 1, grade ≥3 TRAEs were reported in 13 patients and no dose-dependent increase in the incidence and grade of TRAEs was observed (Supplementary Table S3). During part 2 and the expansion part (*N* = 61), the most common TRAEs occurred in more than 30% of patients were increased AST (31patients, 50.8%), fatigue (27 patients, 44.3%), nausea and decreased appetite (26 patients, 42.6% each), increased ALT (23 patients, 37.7%), neutropenia and anemia (21 patients, 34.4% each), hypophosphatemia (20 patients, 32.8%), vomiting and thrombocytopenia (19 patients, 31.1% each). Grade ≥3 TRAEs were reported in 47 patients (77.0%), with neutropenia, hypophosphatemia, anemia, thrombocytopenia, leukocytopenia occurring in ≥10% of patients (Table 2).

**Table 2:** Incidence of treatment-related adverse events in ≥20% of patients in part 1 or part 2 + expansion part

	Part 1		Part 2 + Expansion part	
	200–800 mg/day, SID		450 or 650 mg/day, 5-on/2-off	
	(*N* = 21)		(*N* = 61)	
	Any Grade	≥ Grade 3	Any Grade	≥ Grade 3
Any Events	21 (100.0)	13 (61.9)	60 (98.4)	47 (77.0)
Aspartate aminotransferase increased	15 (71.4)	0 (0.0)	31 (50.8)	4 (6.6)
Rash	12 (57.1)	2 (9.5)	15 (24.6)	1 (1.6)
Alanine aminotransferase increased	11 (52.4)	0 (0.0)	23 (37.7)	1 (1.6)
Fatigue	10 (47.6)	0 (0.0)	27 (44.3)	1 (1.6)
Amylase increased	8 (38.1)	3 (14.3)	10 (16.4)	1 (1.6)
Neutropenia	8 (38.1)	3 (14.3)	21 (34.4)	15 (24.6)
Thrombocytopenia	8 (38.1)	1 (4.8)	19 (31.1)	9 (14.8)
Leukocytopenia	8 (38.1)	2 (9.5)	18 (29.5)	7 (11.5)
Nausea	7 (33.3)	0 (0.0)	26 (42.6)	1 (1.6)
Decreased appetite	7 (33.3)	1 (4.8)	26 (42.6)	6 (9.8)
Face edema	6 (28.6)	0 (0.0)	11 (18.0)	0 (0.0)
Edema	6 (28.6)	0 (0.0)	10 (16.4)	0 (0.0)
Lipase increased	6 (28.6)	5 (23.8)	9 (14.8)	4 (6.6)
Hypophosphatemia	6 (28.6)	5 (23.8)	20 (32.8)	13 (21.3)
Vomiting	4 (19.0)	0 (0.0)	19 (31.1)	1 (1.6)
Diarrhea	3 (14.3)	0 (0.0)	14 (23.0)	0 (0.0)
Anemia	2 (9.5)	2 (9.5)	21 (34.4)	9 (14.8)

*SID* once daily

TRAEs that led to treatment discontinuation were rash (one patient, 800 mg) in part 1, rash (two patients, 650 mg), pyrexia, peritonitis and neutropenia (one patient each, 650 mg) in part 2 and expansion part. All the patients in cohorts 650 mg and 800 mg in part 1 experienced TRAEs leading to dose interruption, while the frequency in part 2 and the expansion part was decreased (approximately 77%). Serious AEs were reported in seven patients in part 1, two patients in part 2 and thirty one patients in the expansion part. Serious TRAEs were tumor hemorrhage and decreased appetite (one patient, 650 mg each), thrombocytopenia (one patient, 800 mg) in part 1, peritonitis (one patient) in part 2, decreased appetite (three patients), nausea (two patients), interstitial lung disease, rash, enterocolitis, fatigue, thrombocytopenia, and pyrexia (one patient each) in the expansion part. AEs resulted in death were hepatic failure, disease progression and respiratory failure occurred in one patient each in the expansion part (650 mg) and this was not considered related to TAS-115. No treatment-related death was observed in any of the study parts during the treatment period or within 28 days of the last dose of TAS-115.

### Antitumor effects

Of the 82 patients in all parts, the best overall response was stable disease (SD) in 31 patients (37.8%), and of these, seventeen patients (8 for osteosarcoma, 4 for prostate cancer and 1 each for bladder cancer, renal cancer, breast cancer, clear cell sarcoma, and gastrointestinal stromal tumor [GIST]), had SD lasting 12 weeks or more. Minor tumor shrinkage of target lesions was reported in four patients who were determined as having progressive disease (PD).

**Fig. 2:**
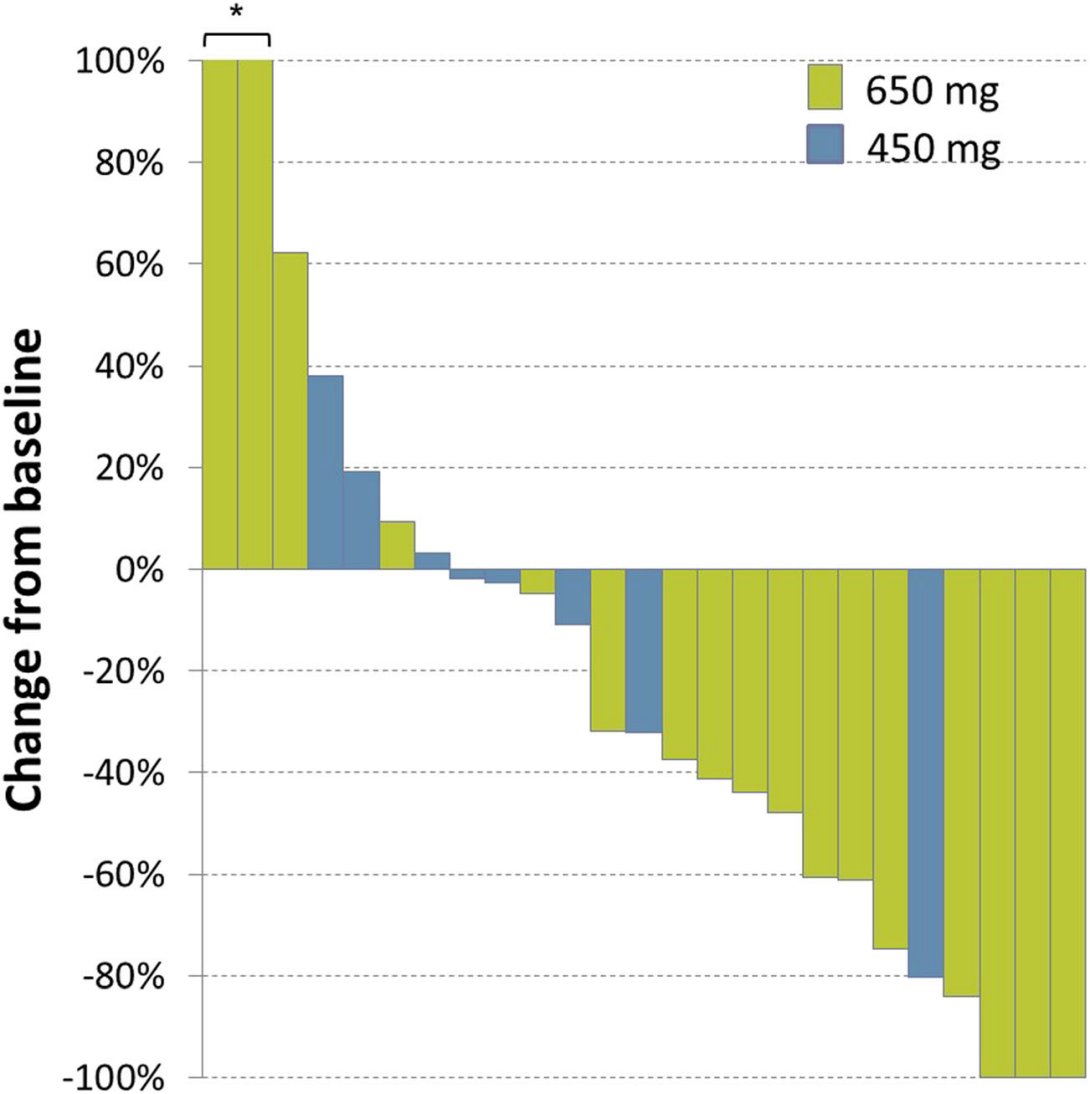
Bone scan index response for best percentage change from baseline in 25 patients enrolled in the expansion part

BSI response rate in patients with bone lesions was 56.0% (14 of 25) among the patients in whom percentage change in BSI was assessed between baseline and at least once post-scan. The primary cancer types of the responders were osteosarcoma in 6 patients, prostate cancer in five patients, epithelioid sarcoma, clear cell sarcoma, and breast cancer in one patient each. The percent change from baseline is shown in the waterfall plots (Fig. 2). The BSI analysis indicated a definite decrease in the quantitative value of primary lesions or metastases to the bone in several patients.

**Fig. 3:**
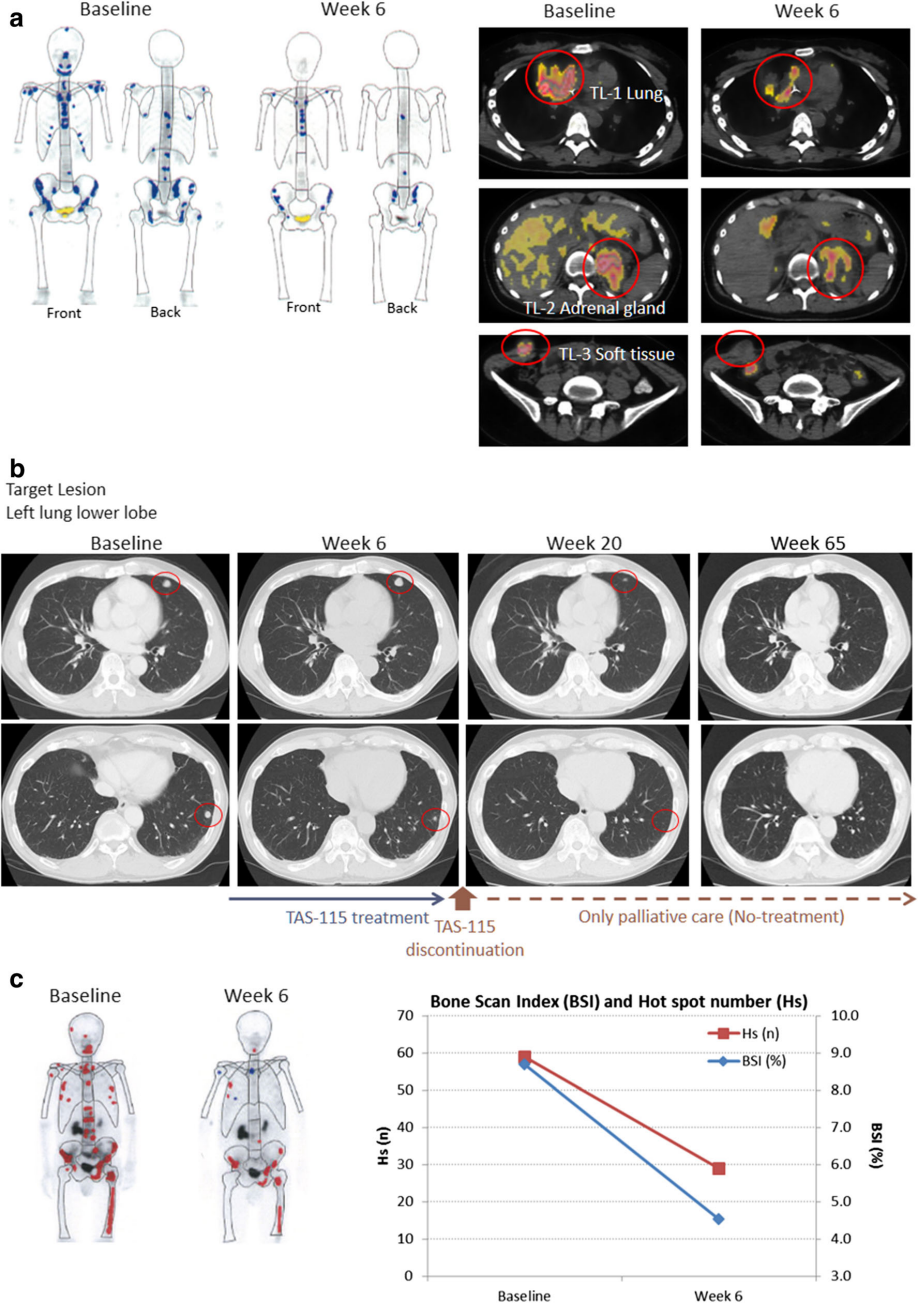
Antitumour effect in **a** a female patient diagnosed with epithelioid sarcoma, **b** a male patient diagnosed with bladder cancer, and **c** a male patient diagnosed with prostate cancer. **a** A female patient in her 20s was initially diagnosed with epithelioid sarcoma. She had received prior treatment for advanced disease with doxorubicin + ifosfamide, carboplatin + etoposide, cisplatin, and pazopanib. At the baseline assessment of this study, she had metastatic disease in bone, lung, adrenal glands, and soft tissue (right-side abdominal). The numbers of hot spots in the bone scan index and fluorodesoxyglucose uptake in the PET-CT scan were reduced at the post-study assessment at Week 6. **b** A male patient in his 70s was diagnosed with bladder cancer. He had received prior neo-adjuvant treatment with gemcitabine+cisplatin (GC), but had no surgery after GC therapy. At the baseline assessment of this study, he had metastases in pleural lymph nodes, regional lymph nodes, and lung. Administration of TAS-115 650 mg 5-on/2-off was discontinued due to the patient’s withdrawal of consent at Week 7, and lung lesions disappeared after 65 weeks. The patient had only palliative care (no systemic anti-cancer therapy) post-TAS-115 treatment. **c** A male patient in his 70s was initially diagnosed with prostate cancer. He had received prior treatment with hormone therapy, abiraterone, docetaxel, cabazitaxel, enzalutamide, darolutamide, and docetaxel again. At the baseline assessment of this study, he had metastatic disease in liver, peritoneum, bone, kidney, and pleural effusion. The bone scan index and the number of hot spots of multiple bone metastases were significantly reduced

Figure 3 shows the representative antitumor effect of TAS-115 as observed in three patients who received 650 mg in a 5-on/2-off schedule. A marked decrease of hot spots on bone scans and reduction in fluorodesoxyglucose uptake were observed 6 weeks after the first administration in a patient with epithelioid sarcoma with multiple metastases (bone, lung, adrenal glands, and soft tissue) (Fig. 3a). In a patient who had bladder cancer with metastases in lung, pleural lymph nodes, and regional lymph nodes, administration of TAS-115 was discontinued at week 7, and the lung metastatic lesions disappeared after discontinuation of TAS-115 treatment without further antitumor treatment (Fig. 3b). In addition, a notable improvement was observed in the bone scan of a patient with CRPC with multiple bone metastases (Fig. 3c).

### Pharmacodynamic profile

A significant increase of sMET in plasma was observed on Day 22 in part 1 (*P* = .0031) and Day 19 (pre-dose and 6 h after TAS-115 administration) in part 2 and the expansion part (*P* = .0015 and *P* = .0055, respectively). At 6 h post-dose on Day 19, an increase of VEGF and decrease of VEGFR2 were observed (*P* = .0844 and *P* = .0204 respectively), as compared with pre-dose on Day 19 (Supplementary Fig. S2). The relationship between biological changes of the protein levels and antitumor activity was not evaluated due to a limited number of patients with clinical effect.

## Discussion

In patients with advanced solid tumors, TAS-115 was generally well tolerated with manageable toxicities. There were no TRAEs that increased dose-dependently in frequency. Although 650 mg SID was determined as the MTD, all patients who received 650 mg SID had treatment interruption due to TRAEs. Thus, a 2-day predefined interruption was included in part 2. Compared with consecutive dosing, the modified dosing schedule led to an improvement in the median relative dose intensity considered to improve recovery from toxicities. Therefore, RP2D was estimated as 650 mg 5-on/2-off for 21 days per cycle.

TAS-115 is similar to cabozantinib in mechanism of action. Cabozantinib inhibits the tyrosine kinase activity of MET, VEGFR and so on, while TAS-115 inhibits FMS and PDGFR in addition to MET and VEGFR. Cabozantinib showed that most common TRAEs (>30% of patients) were diarrhea (57%), fatigue (55%), decreased appetite (48%) and nausea (43%) in phase I study for solid tumours [19]. TAS-115 showed similar TRAEs with cabozantinib, but the frequency of diarrhea in TAS-115 was lower compared to cabozantinib. While, neutropenia, anemia, hypophosphatemia, and thrombocytopenia were not observed in cabozantinib, and these TRAEs are considered TAS-115 specific TRAEs.

PK analyses showed rapid absorption of TAS-115 with a moderate dose-dependent increase in TAS-115 exposure, and no unexpected accumulation was observed in parts 1 and 2. The PK profile showing lack of accumulation and a short half-life may contribute to the tolerability of TAS-115. A dose-dependent increase up to 650 mg in TAS-115 exposure was observed. In preliminary PK analyses of food effect, C_max_ and AUC_0–24_ under fed condition to those under empty stomach condition were 0.545 (0.243–1.224) and 0.812 (0.414–1.593). As there were no statistically significant differences in both parameters, it will be confirmed in further study.

Evaluation of pharmacodynamic markers revealed a significant increase in plasma sMET. Increased levels of circulating sMET were reported in previous clinical studies with cabozantinib, rilotumumab, and onartuzumab [19–21]. The elevated plasma sMET in this study may be associated with MET inhibition following TAS-115 administration. Marked alterations of VEGF and sVEGFR2 from Day 1 were not observed, which may be a result of the short half-life of TAS-115. However, comparing pre- and post-dose on Day 19, a VEGFR inhibitory activity of TAS-115 was suggested.

Although there were no patients who showed complete or partial response by RECIST evaluation, TAS-115 exhibited encouraging antitumor effects on the bone metastases, which is in line with preclinical data of TAS-115. In vitro data showed that TAS-115 caused a dose-dependent inhibition of osteoclast differentiation induced by M-CSF and receptor activator of nuclear factor-kappa B ligand (RANKL) (data on file). TAS-115 also suppressed HGF, and VEGF-induced phosphorylation of signaling factors (AKT and ERK1/2) in mouse osteoclasts in vitro [14, 15]. The decline of BSI assessed using BONENAVI® has been reported to be associated with survival in patients with CRPC [22], and this effect on bone metastases may improve the quality of life of patients [23]. The efficacy of TAS-115 on bone was supported by non-clinical findings of inhibition of aberrant bone remodeling and FMS as well as VEGFRs and MET. In the expansion part, we investigated 450 mg/day dosage in CRPC patients with bone metastases in order to improve the administration continuity and dose intensity in elderly patients. Furthermore, a phase II trial for CRPC patients is currently in progress (JapicCTI-163448). Remarkably, a patient who had bladder cancer with lung metastases showed the disappearance of the lung metastatic lesions during palliative care after 58 weeks from discontinuation of TAS-115 treatment (Fig. 3b). We speculated that this tumor shrinkage was caused by inhibition of CSF-1R in macrophages with TAS-115. Inhibition of CSF-1R by TAS-115 may suppress the polarization into M2 macrophages that depends on CSF-1 signaling, which promotes tumor growth, invasion, and metastasis, and thus, the immune environment in tumors may be improved.

In conclusion, TAS-115 was generally well tolerated with manageable toxicities at dose levels of up to and including 650 mg on a 5-on/2-off schedule. From safety and PK profile, RP2D was estimated as 650 mg SID, 5-on/2-off. Pharmacodynamic results demonstrated that TAS-115 acts as a multi-kinase inhibitor. Furthermore, antitumor efficacy, particularly in patients with bone metastases, was encouraging and warrants further investigation. A phase II study was started in patients with castration-resistant prostate cancer with bone metastases.

## Electronic supplementary material

ESM 1 (PDF 370 kb)
