# Supplementary material for: Phase I study of TAS-115, a novel oral multi-kinase inhibitor, in patients with advanced solid tumors
Source: Invest New Drugs. 2019 Dec 10;38(4):1175–85. doi: 10.1007/s10637-019-00859-4 (PMC7340670; doi:10.1007/s10637-019-00859-4)
Supplement: Supplementary file 1 — (PDF 370 kb) [file 10637_2019_859_MOESM1_ESM.pdf]

Supplemental Information

Fig S1. Study design, including dose-escalation schematic.

SID once daily, *MTD* maximum tolerated dose, *CRPC* castration-resistant prostate cancer.

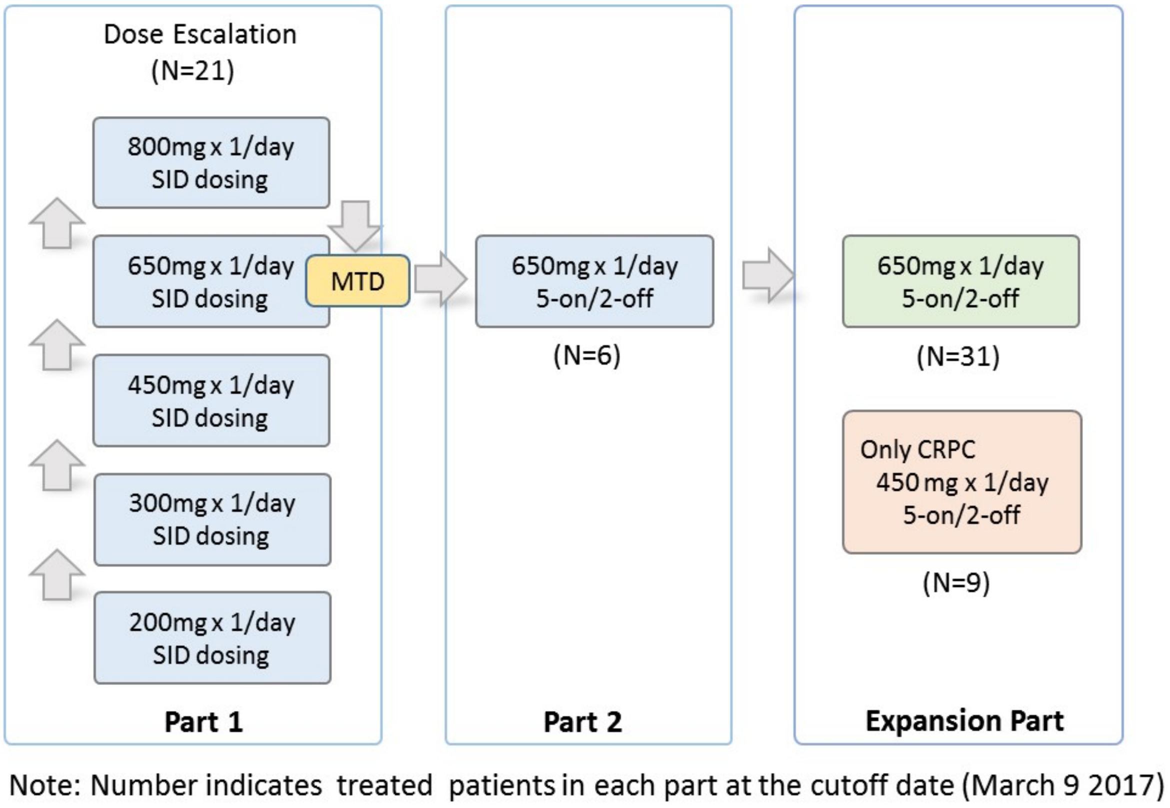

Fig S2. Pharmacodynamic markers. Change from baseline and post TAS-115 treatment in A) soluble MET, B) HGF, C) soluble VEGFR2, and D) VEGF.

MET hepatocyte growth factor (HGF) receptor, *sMET* soluble MET, *VEGF* vascular endothelial growth factor, *VEGFR* VEGF receptor, *sVEGFR* soluble VEGFR.

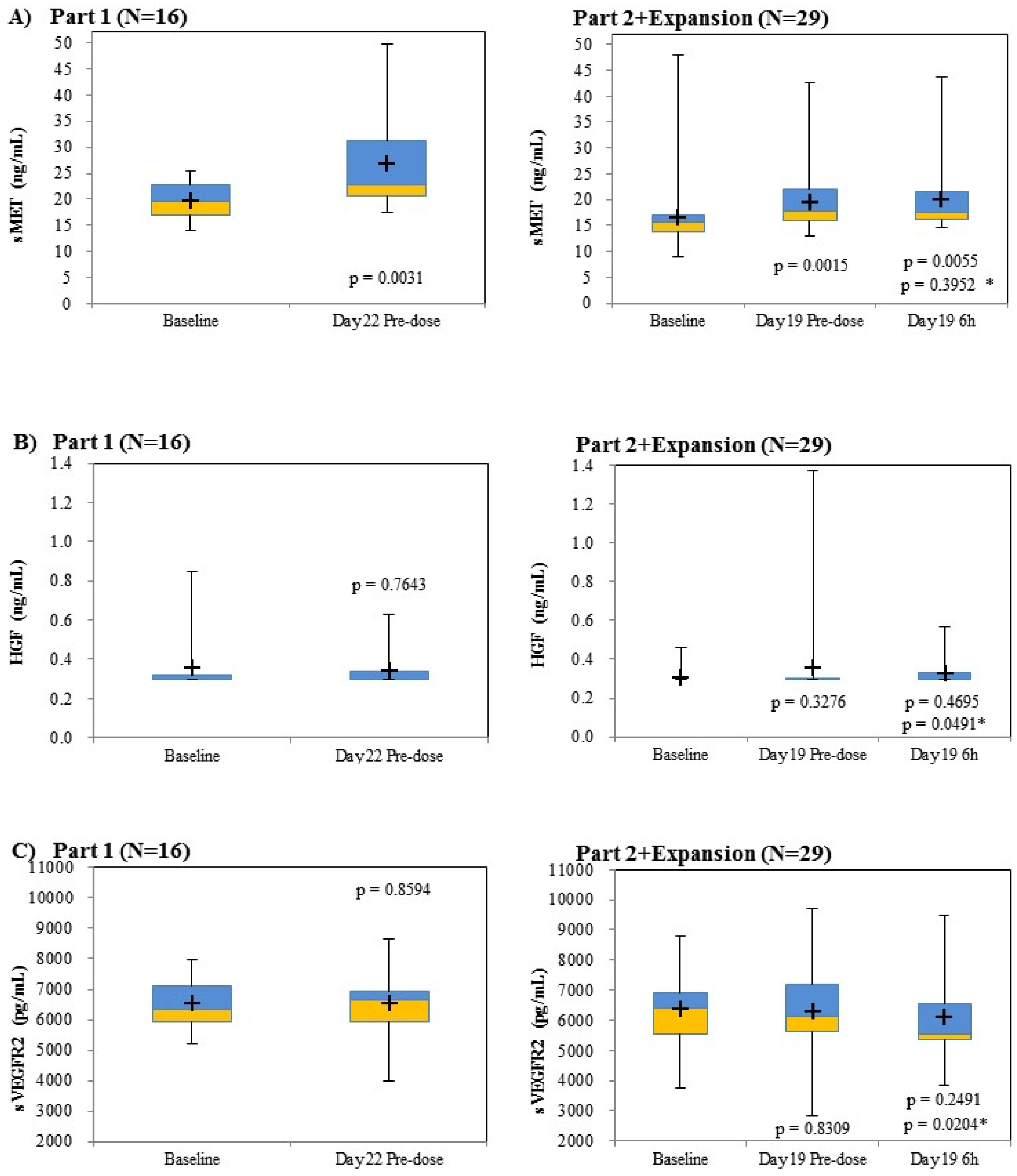

D)

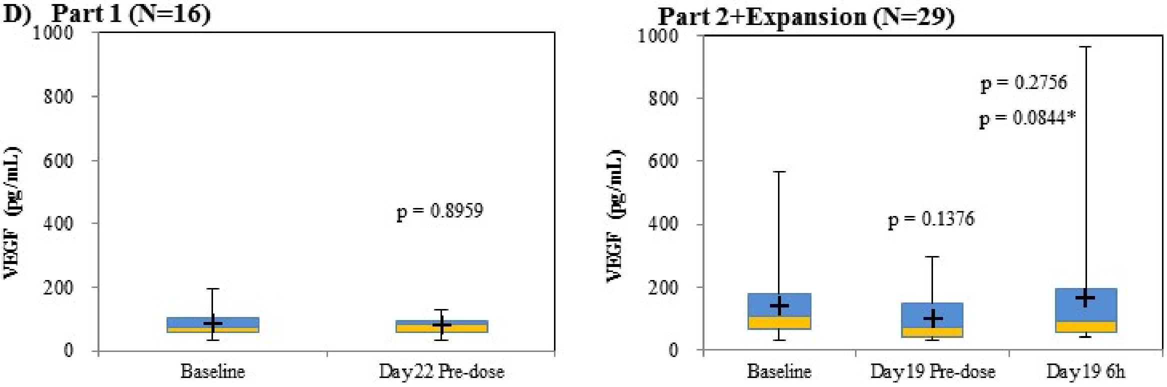

Significant at *P* < .05

* Day19 Pre-dose vs Day 19 6h

Table S1. Dose resumption and elevation criteria

**Resumption criteria**	**Adverse event**	**Criteria**
	Neutrophil count[1]	≥ 1,500/mm^3
	Platelet count[2]	≥ 75,000/mm^3
	Non-hematotoxicity	Return to baseline or Grade ≤ 2
	Other	Adverse events leading to the decision of drug interruption are alleviated or resolved
**Criteria for dose elevation from the initial dose**	The dose can be increased in patients before MTD is determined at each part. From cycle 2 onward, if any patients are receiving a dose ≥2 levels lower than patients receiving the highest dose among the study patients, the dose for those patients can be increased to a dose one level lower than that of patients receiving the highest dose (e.g., if a patient's dose is escalated to a level 4 dose, all level 1 and 2 dose patients can be escalated to a level 3 dose). However, dose escalation should be performed at Day 1 of each cycle.	
**Criteria for dose elevation after dose reduction**	In patients in whom the dose is reduced due to causes other than DLT, the dose could be escalated back to the original dose if the safety concern that led to dose reduction is determined to have been resolved by the investigator/sub-investigator.	

[1] For neutrophil count, the test value recorded at ≥48 hours after administration of G-CSF preparations meets the criterion.
[2] For platelet count, the test value recorded at ≥48 after transfusion meets the criterion.

DLT dose-limiting toxicity, *G-CSF*, granulocyte colony-stimulating factor; *MTD* maximum tolerated dose.

Table S2. Pharmacokinetic parameters

Part	Day/Condition	Dose (mg/day)	n	C_{max} (ng/mL) Mean (SD)	t_{max} (h) Median	AUC_{0-24} (ng·hr/mL) Mean (SD)	$t_{1/2}$ (h) Mean (SD)
Part 1	Day 1 (Single administration)	200	3*	2493 (600)	1.0	19963 (4185)	15.2 (NC)
		300	3	3503 (2592)	1.0	23217 (6564)	20.4 (9.4)
		450	3	4537 (2140)	1.0	28829 (11456)	12.6 (8.4)
		650	6	7703 (5517)	1.0	59668 (28482)	10.3 (4.6)
		800	6	4912 (3019)	1.0	37754 (12440)	13.7 (7.8)
	Day 8 (Multiple administration)	200	3	2950 (830)	1.0	22655 (6301)	26.6 (27.4)
		300	2	3905 (NC)	1.5	32530 (NC)	15.9 (NC)
		450	3	4313 (1202)	1.0	36980 (13874)	11.4 (8.2)
		650	5†	5038 (1194)	2.0	54544 (27672)	8.7 (1.5)
		800	6	5220 (2918)	1.5	44634 (20313)	12.8 (3.9)
Part 2	Day 1 (Single administration)	650	6#	7512 (2602)	1.0	49785 (17638)	14.5 (8.6)
	Day 19 (Multiple administration)	650	6	7372 (2286)	1.0	56661 (22333)	16.8 (10.3)
Expansion part	Fed	650	6	3905 (3694)	5.0	41667 (31388)	11.7 (3.6)
	Empty Stomach	650	6	4755 (2797)	2.0	39251 (21275)	11.4 (2.7)

* For $t_{1/2}$, n=2
† For $t_{1/2}$, n=4
# For $t_{1/2}$, n=5

The PK analyses of TAS-115 were conducted with validated liquid chromatography-tandem mass spectrometry. PK parameters were calculated by non-compartmental analysis with Phoenix® WinNonlin® version 6.4 software (Certara LP, Princeton, NJ, USA).

AUC area under the time-concentration curve from 24 hours [AUC_{0-24}], C_{max} maximum plasma concentration, *NC* not calculated, *SD* standard deviation, t_{max} time to maximum concentration, $t_{1/2}$ elimination half-life.

Table S3. Treatment-related adverse events by dose level in part 1 (N=21)

	200 mg SID (N=3)		300 mg SID (N=3)		450 mg SID (N=3)		650 mg SID (N=6)		800 mg SID (N=6)	
	Total N (%)	≥ Grade 3 N (%)	Total N (%)	≥ Grade 3 N (%)	Total N (%)	≥ Grade 3 N (%)	Total N (%)	≥ Grade 3 N (%)	Total N (%)	≥ Grade 3 N (%)
**Any Events**	**3 (100.0)**	**1 (33.3)**	**3 (100.0)**	**2 (66.7)**	**3 (100.0)**	**2 (66.7)**	**6 (100.0)**	**5 (83.3)**	**6 (100.0)**	**3 (50.0)**
**Blood and lymphatic system disorders**			**1 (33.3)**	**1 (33.3)**			**1 (16.7)**	**1 (16.7)**		
Anaemia			1 (33.3)	1 (33.3)			1 (16.7)	1 (16.7)		
**Ear and labyrinth disorders**			**1 (33.3)**							
Vertigo			1 (33.3)							
**Eye disorders**							**1 (16.7)**			
Conjunctival haemorrhage							1 (16.7)			
**Gastrointestinal disorders**	**1 (33.3)**		**3 (100.0)**				**5 (83.3)**		**4 (66.7)**	
Abdominal pain							1 (16.7)			
Abdominal pain upper							1 (16.7)			
Cheilitis							1 (16.7)			
Diarrhoea			2 (66.7)				1 (16.7)			
Nausea	1 (33.3)		1 (33.3)				4 (66.7)		1 (16.7)	
Stomatitis							1 (16.7)		2 (33.3)	
Vomiting	1 (33.3)		1 (33.3)						2 (33.3)	
**General disorders and administration site conditions**	**3 (100.0)**		**3 (100.0)**		**3 (100.0)**		**2 (33.3)**		**5 (83.3)**	
Face oedema	2 (66.7)		1 (33.3)						3 (50.0)	
Fatigue	1 (33.3)		2 (66.7)		1 (33.3)		2 (33.3)		4 (66.7)	
Mucosal inflammation							1 (16.7)			
Oedema	1 (33.3)		1 (33.3)		3 (100.0)		1 (16.7)			
Pyrexia			1 (33.3)						1 (16.7)	
**Investigations**	**2 (66.7)**	**1 (33.3)**	**2 (66.7)**	**2 (66.7)**	**3 (100.0)**	**2 (66.7)**	**6 (100.0)**	**3 (50.0)**	**4 (66.7)**	**1 (16.7)**
Alanine aminotransferase increased			2 (66.7)		1 (33.3)		4 (66.7)		4 (66.7)	
Amylase increased	1 (33.3)	1 (33.3)	1 (33.3)		1 (33.3)		3 (50.0)	1 (16.7)	2 (33.3)	1 (16.7)
Aspartate aminotransferase increased	1 (33.3)		2 (66.7)		2 (66.7)		6 (100.0)		4 (66.7)	
Beta 2 microglobulin urine increased			1 (33.3)						1 (16.7)	
Blood corticotrophin decreased									1 (16.7)	
Blood creatine phosphokinase increased			1 (33.3)				1 (16.7)		1 (16.7)	
Blood creatinine increased					1 (33.3)					
Blood fibrinogen decreased			1 (33.3)		1 (33.3)					
Blood thyroid stimulating hormone decreased									1 (16.7)	
Electrocardiogram QT prolonged							1 (16.7)	1 (16.7)		
Fibrin D dimer increased			1 (33.3)		1 (33.3)		1 (16.7)			

	**200 mg SID (N=3)**		**300 mg SID (N=3)**		**450 mg SID (N=3)**		**650 mg SID (N=6)**		**800 mg SID (N=6)**	
	Total N (%)	≥ Grade 3 N (%)	Total N (%)	≥ Grade 3 N (%)	Total N (%)	≥ Grade 3 N (%)	Total N (%)	≥ Grade 3 N (%)	Total N (%)	≥ Grade 3 N (%)
Gamma-glutamyltransferase increased			1 (33.3)							
Lipase increased	1 (33.3)	1 (33.3)	1 (33.3)	1 (33.3)	1 (33.3)	1 (33.3)	2 (33.3)	2 (33.3)	1 (16.7)	
Neutrophil count decreased	1 (33.3)		2 (66.7)	1 (33.3)	2 (66.7)	1 (33.3)	1 (16.7)		2 (33.3)	1 (16.7)
Platelet count decreased	1 (33.3)		2 (66.7)		1 (33.3)		2 (33.3)		2 (33.3)	1 (16.7)
White blood cell count decreased	1 (33.3)		2 (66.7)	1 (33.3)	2 (66.7)		1 (16.7)		2 (33.3)	1 (16.7)
Blood alkaline phosphatase increased									1 (16.7)	
**Metabolism and nutrition disorders**			**2 (66.7)**	**1 (33.3)**	**2 (66.7)**	**1 (33.3)**	**4 (66.7)**	**1 (16.7)**	**4 (66.7)**	**2 (33.3)**
Hypoalbuminaemia					1 (33.3)					
Hypokalaemia			1 (33.3)	1 (33.3)						
Hypophosphataemia			1 (33.3)	1 (33.3)	1 (33.3)	1 (33.3)	1 (16.7)	1 (16.7)	3 (50.0)	2 (33.3)
Decreased appetite			1 (33.3)				4 (66.7)		2 (33.3)	1 (16.7)
**Musculoskeletal and connective tissue disorders**							**1 (16.7)**			
Arthralgia							1 (16.7)			
**Neoplasms benign, malignant and unspecified (incl cysts and polyps)**							**1 (16.7)**			
Tumour haemorrhage							1 (16.7)			
**Nervous system disorders**							**1 (16.7)**		**1 (16.7)**	
Dysgeusia							1 (16.7)			
Peripheral sensory neuropathy									1 (16.7)	
**Renal and urinary disorders**			**2 (66.7)**				**1 (16.7)**			
Proteinuria			2 (66.7)				1 (16.7)			
**Reproductive system and breast disorders**	**1 (33.3)**									
Vaginal haemorrhage	1 (33.3)									
**Skin and subcutaneous tissue disorders**	**2 (66.7)**		**1 (33.3)**		**1 (33.3)**		**5 (83.3)**	**1 (16.7)**	**3 (50.0)**	**1 (16.7)**
Palmar-plantar erythrodysaesthesia syndrome							2 (33.3)			
Pruritus							1 (16.7)			
Rash	2 (66.7)		1 (33.3)		1 (33.3)		5 (83.3)	1 (16.7)	3 (50.0)	1 (16.7)
**Vascular disorders**			**1 (33.3)**				**1 (16.7)**		**1 (16.7)**	**1 (16.7)**
Hypertension			1 (33.3)				1 (16.7)		1 (16.7)	1 (16.7)

SID once daily.
